# Tetra­kis[3-(2-pyridylamino)pyridine-κ*N*]nickel(II) diperchlorate ethanol disolvate

**DOI:** 10.1107/S1600536808006569

**Published:** 2008-03-14

**Authors:** Zhi-Min Wang

**Affiliations:** a College of Biology and Environment Engineering, Zhejiang Shuren University, Hangzhou 310015, People’s Republic of China

## Abstract

In the title compound, [Ni(C_10_H_9_N_3_)_4_](ClO_4_)_2_·2C_2_H_5_OH, the metal centre exhibits a four-coordinated environment with four pyridine N atoms of the four different dipyridylamine ligands. A twofold rotation axis passes through the Ni atom. N—H⋯O and N—H⋯N hydrogen bonds are present in the crystal structure.

## Related literature

For related literature, see: Moulton & Zaworotko (2001 ▶); Su *et al.* (2003 ▶); Zhou *et al.* (2006 ▶); Biradha *et al.* (1999 ▶); Gudbjartson *et al.* (1999 ▶). 
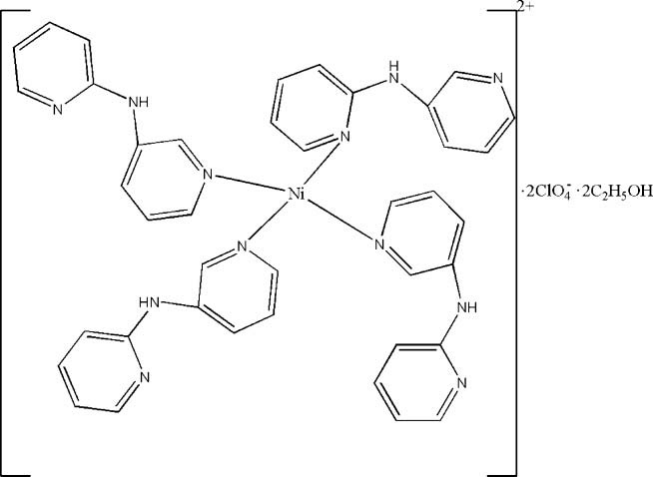


## Experimental

### 

#### Crystal data

[Ni(C_10_H_9_N_3_)_4_](ClO_4_)_2_·2C_2_H_6_O
*M* *_r_* = 1034.55
Monoclinic, 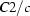

*a* = 27.767 (4) Å
*b* = 10.7067 (14) Å
*c* = 18.144 (2) Å
β = 115.891 (9)°
*V* = 4852.6 (11) Å^3^
*Z* = 4
Mo *K*α radiation
μ = 0.58 mm^−1^
*T* = 298 (2) K
0.32 × 0.22 × 0.17 mm

#### Data collection

Bruker APEXII area-detector diffractometer
Absorption correction: multi-scan (*SADABS*; Sheldrick, 2004 ▶) *T*_min_ = 0.836, *T*_max_ = 0.908
11042 measured reflections
4315 independent reflections
2875 reflections with *I* > 2σ(*I*)
*R*_int_ = 0.044

#### Refinement

*R*[*F* ^2^ > 2σ(*F* ^2^)] = 0.048
*wR*(*F* ^2^) = 0.134
*S* = 1.08
4315 reflections
314 parameters
H-atom parameters constrained
Δρ_max_ = 0.35 e Å^−3^
Δρ_min_ = −0.36 e Å^−3^

### 

Data collection: *APEX2* (Bruker, 2004 ▶); cell refinement: *SAINT* (Bruker, 2004 ▶); data reduction: *SAINT*; program(s) used to solve structure: *SHELXS97* (Sheldrick, 2008 ▶); program(s) used to refine structure: *SHELXL97* (Sheldrick, 2008 ▶); molecular graphics: *SHELXTL* (Sheldrick, 2008 ▶); software used to prepare material for publication: *SHELXTL*.

## Supplementary Material

Crystal structure: contains datablocks I, global. DOI: 10.1107/S1600536808006569/at2548sup1.cif

Structure factors: contains datablocks I. DOI: 10.1107/S1600536808006569/at2548Isup2.hkl

Additional supplementary materials:  crystallographic information; 3D view; checkCIF report

## Figures and Tables

**Table 1:** Hydrogen-bond geometry (Å, °)

*D*—H⋯*A*	*D*—H	H⋯*A*	*D*⋯*A*	*D*—H⋯*A*
N5—H5*B*⋯O5	0.86	2.07	2.928 (5)	174
N2—H2⋯N6^i^	0.86	2.27	3.129 (4)	176

Symmetry code: (i) 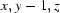.

**Fig. 1.**
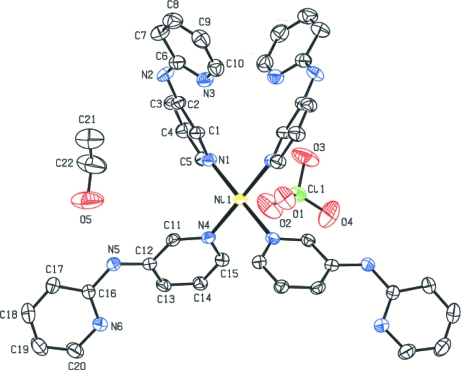
The asymmetric unit of (I), showing 30% probability displacement ellipsoids [symmetrical code: (i) -x, y, 1/2 - z].

## supplementary crystallographic information

### Comment

In recent years, the rational design and assembly of metal-organic frameworks (MOFs) with well regulated network structures have received remarkable attention in order to develop new functional materials with potential applications (Moulton & Zaworotko, 2001). Nevertheless, it is still a great challenge to predict the exact structures and compositions of polymeric compounds assembled in a helical motif, although some structures with various helices have been reported in MOFs. So far, much of the research has been concentrated on the exploitation of angular ligands with a molecular angle, such as ligands with a T-shape, V-shape *etc*, in the construction of versatile coordination polymer architectures (Gudbjartson *et al.*, 1999; Su *et al.*, 2003, Zhou *et al.*, 2006). In this paper, we report the synthesis and crystal structure of the title complex with a V-shaped ligand,(I).

As shown in Fig. 1, the complex I is located twofold axis *via* the 2,3'-dipyridylamine (*L*) ligands. That I is a neutral, mononuclear molecule with the Ni(II) atom in a square coordination geometry with four pyridine nitrogen atoms of the four different *L* ligands. The Ni—N bond lengths range from 2.013 (2) to 2.019 (2)Å (Table1), and the N(1)—Ni—N(4) angle is 179.09 (11)°, it can be seen that the Ni(II) ions together with the four nitrogen atoms form a perfect square geometry, and this ideal quadrangle structure is rare in the coordination geometry of Ni(II) atom. Four *L* ligands present monodenate fashion. Two O atoms of the uncoordinated ClO_4_ anions form the acceptors of intermolecular hydrogen bonds and weak interactions, which link the discrete units to form a two-dimensional supramolecular structure. The ethanol molecule present in I only functioned as an acceptor of intramolecular hydrogen bonds between the oxygen atoms of ethonal and amino nitrogen atoms of the ligand (Table 2), which stabilize the extended structure.

### Experimental

NiClO_4 _(0.027 g, 0.013 mmol), *L* (0.025 g, 0.014 mmol) were added in a solvent of methanol, the mixture was heated for 6 h under reflux. During the process stirring and influx were required. The resultant was then filtered to give a pure solution which was infiltrated by diethyl ether freely in a closed vessel, Two weeks later some single crystals of the size suitable for X-Ray diffraction analysis.

### Refinement

The H atoms (pyridine ring) were placed in calculated positions [C—H = 0.93 - 0.97 Å and N—H = 0.86 Å] and refined using a riding model, with *U*_iso_(H) = 1.2*U*_eq_(C,*N*) or 1.5*U*_eq_(C_methyl_).

### Crystal data

**Table:**

[Ni(C_10_H_9_N_3_)_4_](ClO_4_)_2_·2C_2_H_6_O	*F*_000_ = 2152
*M**_r_* = 1034.55	*D*_x_ = 1.416 Mg m^−^^3^
Monoclinic, *C*2/*c*	Mo *K*α radiation λ = 0.71073 Å
Hall symbol: -C 2yc	Cell parameters from 4315 reflections
*a* = 27.767 (4) Å	θ = 1.6–25.1º
*b* = 10.7067 (14) Å	µ = 0.58 mm^−^^1^
*c* = 18.144 (2) Å	*T* = 298 (2) K
β = 115.891 (9)º	Block, green
*V* = 4852.6 (11) Å^3^	0.32 × 0.22 × 0.17 mm
*Z* = 4	

### Data collection

**Table:**

Bruker APEXII area-detector diffractometer	4315 independent reflections
Radiation source: fine-focus sealed tube	2875 reflections with *I* > 2σ(*I*)
Monochromator: graphite	*R*_int_ = 0.044
*T* = 298(2) K	θ_max_ = 25.1º
φ and ω scans	θ_min_ = 1.6º
Absorption correction: multi-scan(SADABS; Sheldrick, 2004)	*h* = −33→33
*T*_min_ = 0.836, *T*_max_ = 0.908	*k* = −12→12
11042 measured reflections	*l* = −21→21

### Refinement

**Table:**

Refinement on *F*^2^	Secondary atom site location: difference Fourier map
Least-squares matrix: full	Hydrogen site location: inferred from neighbouring sites
*R*[*F*^2^ > 2σ(*F*^2^)] = 0.048	H-atom parameters constrained
*wR*(*F*^2^) = 0.134	*w* = 1/[σ^2^(*F*_o_^2^) + (0.0501*P*)^2^ + 1.1196*P*] where *P* = (*F*_o_^2^ + 2*F*_c_^2^)/3
*S* = 1.08	(Δ/σ)_max_ = 0.084
4315 reflections	Δρ_max_ = 0.35 e Å^−^^3^
314 parameters	Δρ_min_ = −0.36 e Å^−^^3^
Primary atom site location: structure-invariant direct methods	Extinction correction: none

### Special details

**Table:**

Geometry. All e.s.d.'s (except the e.s.d. in the dihedral angle between two l.s. planes) are estimated using the full covariance matrix. The cell e.s.d.'s are taken into account individually in the estimation of e.s.d.'s in distances, angles and torsion angles; correlations between e.s.d.'s in cell parameters are only used when they are defined by crystal symmetry. An approximate (isotropic) treatment of cell e.s.d.'s is used for estimating e.s.d.'s involving l.s. planes.
Refinement. Refinement of *F*^2^ against ALL reflections. The weighted *R*-factor *wR* and goodness of fit *S* are based on *F*^2^, conventional *R*-factors *R* are based on *F*, with *F* set to zero for negative *F*^2^. The threshold expression of *F*^2^ > σ(*F*^2^) is used only for calculating *R*-factors(gt) *etc*. and is not relevant to the choice of reflections for refinement. *R*-factors based on *F*^2^ are statistically about twice as large as those based on *F*, and *R*- factors based on ALL data will be even larger.

### Fractional atomic coordinates and isotropic or equivalent isotropic displacement parameters (Å^2^)

**Table:**

	*x*	*y*	*z*	*U*_iso_*/*U*_eq_	
Ni1	0.5000	0.23720 (5)	0.7500	0.0397 (2)	
N1	0.54118 (10)	0.1032 (2)	0.72421 (16)	0.0455 (6)	
N2	0.60983 (11)	−0.1938 (3)	0.80972 (17)	0.0586 (8)	
H2	0.6337	−0.2349	0.8016	0.070*	
N3	0.56191 (11)	−0.1786 (3)	0.88745 (18)	0.0577 (7)	
N4	0.54239 (10)	0.3700 (2)	0.72509 (16)	0.0438 (6)	
N5	0.68140 (10)	0.4606 (3)	0.79766 (17)	0.0550 (7)	
H5B	0.6945	0.3923	0.8237	0.066*	
N6	0.69860 (11)	0.6684 (3)	0.77848 (18)	0.0574 (7)	
C1	0.56303 (12)	0.0085 (3)	0.77601 (19)	0.0456 (8)	
H1	0.5621	0.0104	0.8267	0.055*	
C2	0.58706 (13)	−0.0930 (3)	0.7574 (2)	0.0474 (8)	
C3	0.58891 (14)	−0.0912 (3)	0.6822 (2)	0.0547 (9)	
H3	0.6051	−0.1566	0.6677	0.066*	
C4	0.56711 (14)	0.0061 (3)	0.6297 (2)	0.0565 (9)	
H4	0.5684	0.0073	0.5794	0.068*	
C5	0.54302 (13)	0.1029 (3)	0.6514 (2)	0.0515 (8)	
H5	0.5279	0.1687	0.6152	0.062*	
C6	0.59913 (15)	−0.2364 (3)	0.8729 (2)	0.0544 (9)	
C7	0.62701 (16)	−0.3403 (4)	0.9173 (2)	0.0682 (11)	
H7	0.6529	−0.3791	0.9058	0.082*	
C8	0.61504 (17)	−0.3838 (4)	0.9787 (2)	0.0757 (11)	
H8	0.6324	−0.4540	1.0089	0.091*	
C9	0.57742 (17)	−0.3231 (4)	0.9951 (2)	0.0720 (11)	
H9	0.5692	−0.3502	1.0369	0.086*	
C10	0.55247 (15)	−0.2224 (4)	0.9489 (2)	0.0643 (10)	
H10	0.5272	−0.1812	0.9606	0.077*	
C11	0.59564 (12)	0.3773 (3)	0.76688 (19)	0.0437 (7)	
H11	0.6127	0.3229	0.8106	0.052*	
C12	0.62665 (12)	0.4614 (3)	0.74861 (19)	0.0426 (7)	
C13	0.60115 (12)	0.5367 (3)	0.6812 (2)	0.0477 (8)	
H13	0.6208	0.5904	0.6645	0.057*	
C14	0.54629 (13)	0.5316 (3)	0.6387 (2)	0.0525 (8)	
H14	0.5285	0.5841	0.5942	0.063*	
C15	0.51777 (13)	0.4489 (3)	0.6622 (2)	0.0515 (8)	
H15	0.4806	0.4475	0.6339	0.062*	
C16	0.71742 (12)	0.5563 (3)	0.8097 (2)	0.0480 (8)	
C17	0.77153 (13)	0.5330 (4)	0.8563 (2)	0.0653 (10)	
H17	0.7835	0.4542	0.8784	0.078*	
C18	0.80698 (16)	0.6289 (5)	0.8690 (3)	0.0827 (13)	
H18	0.8434	0.6163	0.9007	0.099*	
C19	0.78839 (18)	0.7437 (4)	0.8349 (3)	0.0815 (13)	
H19	0.8118	0.8094	0.8415	0.098*	
C20	0.73503 (16)	0.7584 (4)	0.7913 (3)	0.0701 (11)	
H20	0.7226	0.8366	0.7686	0.084*	
Cl1	0.39899 (4)	0.22782 (8)	0.51603 (5)	0.0580 (3)	
O1	0.42456 (13)	0.2385 (3)	0.60132 (16)	0.0903 (10)	
O2	0.43498 (15)	0.2706 (3)	0.4854 (2)	0.1180 (13)	
O3	0.38628 (16)	0.1017 (3)	0.4956 (2)	0.1268 (14)	
O4	0.35263 (17)	0.2973 (5)	0.4863 (4)	0.196 (2)	
O5	0.7269 (3)	0.2376 (3)	0.8974 (3)	0.166 (2)	
H5A	0.7075	0.2571	0.9190	0.249*	
C21	0.7270 (3)	0.0252 (6)	0.9344 (4)	0.143 (2)	
H21A	0.7532	0.0553	0.9863	0.215*	
H21B	0.7396	−0.0506	0.9206	0.215*	
H21C	0.6940	0.0094	0.9378	0.215*	
C22	0.7186 (3)	0.1150 (6)	0.8741 (4)	0.151 (3)	
H22A	0.7416	0.0948	0.8482	0.181*	
H22B	0.6819	0.1068	0.8327	0.181*	

### Atomic displacement parameters (Å^2^)

**Table:**

	*U*^11^	*U*^22^	*U*^33^	*U*^12^	*U*^13^	*U*^23^
Ni1	0.0470 (3)	0.0271 (3)	0.0562 (4)	0.000	0.0329 (3)	0.000
N1	0.0539 (16)	0.0367 (15)	0.0564 (16)	0.0018 (12)	0.0340 (14)	0.0022 (13)
N2	0.070 (2)	0.0493 (17)	0.0644 (19)	0.0226 (15)	0.0365 (17)	0.0081 (15)
N3	0.0660 (19)	0.0489 (18)	0.0645 (19)	0.0046 (14)	0.0344 (17)	0.0068 (15)
N4	0.0470 (16)	0.0328 (14)	0.0556 (16)	0.0043 (12)	0.0261 (14)	0.0038 (13)
N5	0.0428 (16)	0.0420 (16)	0.071 (2)	0.0045 (12)	0.0166 (15)	0.0154 (14)
N6	0.0506 (17)	0.0438 (18)	0.073 (2)	−0.0016 (14)	0.0221 (16)	0.0044 (15)
C1	0.057 (2)	0.0342 (17)	0.0531 (19)	0.0017 (15)	0.0311 (17)	0.0019 (16)
C2	0.0508 (19)	0.0390 (18)	0.058 (2)	0.0035 (15)	0.0285 (17)	−0.0007 (16)
C3	0.065 (2)	0.046 (2)	0.064 (2)	0.0063 (17)	0.0384 (19)	−0.0056 (18)
C4	0.072 (2)	0.052 (2)	0.059 (2)	0.0061 (18)	0.041 (2)	0.0009 (18)
C5	0.063 (2)	0.046 (2)	0.058 (2)	0.0033 (16)	0.0376 (18)	0.0063 (17)
C6	0.061 (2)	0.044 (2)	0.054 (2)	0.0017 (17)	0.0212 (18)	−0.0013 (17)
C7	0.079 (3)	0.054 (2)	0.068 (3)	0.017 (2)	0.029 (2)	0.011 (2)
C8	0.086 (3)	0.056 (3)	0.065 (3)	0.003 (2)	0.014 (2)	0.012 (2)
C9	0.075 (3)	0.075 (3)	0.059 (2)	−0.012 (2)	0.023 (2)	0.014 (2)
C10	0.061 (2)	0.069 (3)	0.065 (2)	−0.0037 (19)	0.029 (2)	0.006 (2)
C11	0.045 (2)	0.0372 (18)	0.0494 (19)	0.0069 (14)	0.0213 (16)	0.0081 (15)
C12	0.0455 (18)	0.0325 (16)	0.0498 (18)	0.0019 (13)	0.0208 (16)	0.0001 (14)
C13	0.051 (2)	0.0383 (18)	0.054 (2)	−0.0073 (15)	0.0230 (17)	0.0052 (16)
C14	0.048 (2)	0.0414 (19)	0.057 (2)	−0.0002 (15)	0.0123 (17)	0.0124 (16)
C15	0.0446 (19)	0.0412 (19)	0.063 (2)	−0.0004 (15)	0.0184 (17)	0.0032 (17)
C16	0.0405 (18)	0.047 (2)	0.056 (2)	−0.0021 (15)	0.0204 (16)	−0.0038 (16)
C17	0.043 (2)	0.061 (2)	0.079 (3)	0.0068 (17)	0.0141 (19)	0.003 (2)
C18	0.043 (2)	0.095 (4)	0.101 (3)	−0.008 (2)	0.024 (2)	−0.006 (3)
C19	0.064 (3)	0.077 (3)	0.096 (3)	−0.026 (2)	0.029 (3)	−0.005 (3)
C20	0.067 (3)	0.054 (2)	0.078 (3)	−0.0135 (19)	0.022 (2)	0.006 (2)
Cl1	0.0655 (6)	0.0541 (6)	0.0529 (5)	−0.0062 (4)	0.0243 (5)	−0.0040 (4)
O1	0.104 (2)	0.120 (3)	0.0521 (16)	−0.0282 (18)	0.0392 (17)	−0.0173 (15)
O2	0.150 (3)	0.146 (3)	0.084 (2)	−0.053 (2)	0.076 (2)	0.000 (2)
O3	0.183 (4)	0.082 (2)	0.152 (3)	−0.060 (2)	0.106 (3)	−0.062 (2)
O4	0.095 (3)	0.162 (4)	0.274 (6)	0.057 (3)	0.026 (4)	0.053 (4)
O5	0.221 (6)	0.067 (2)	0.128 (4)	0.010 (3)	0.001 (3)	0.019 (2)
C21	0.189 (7)	0.092 (4)	0.147 (6)	−0.001 (4)	0.072 (5)	0.004 (4)
C22	0.187 (7)	0.080 (4)	0.129 (5)	−0.028 (4)	0.017 (5)	0.013 (4)

### Geometric parameters (Å, °)

**Table:**

Ni1—N1^i^	2.013 (2)	C9—C10	1.356 (5)
Ni1—N1	2.013 (2)	C9—H9	0.9300
Ni1—N4	2.019 (2)	C10—H10	0.9300
Ni1—N4^i^	2.019 (2)	C11—C12	1.382 (4)
N1—C1	1.335 (4)	C11—H11	0.9300
N1—C5	1.344 (4)	C12—C13	1.375 (4)
N2—C6	1.382 (4)	C13—C14	1.375 (4)
N2—C2	1.393 (4)	C13—H13	0.9300
N2—H2	0.8600	C14—C15	1.373 (4)
N3—C6	1.326 (4)	C14—H14	0.9300
N3—C10	1.336 (4)	C15—H15	0.9300
N4—C11	1.337 (4)	C16—C17	1.388 (5)
N4—C15	1.342 (4)	C17—C18	1.371 (5)
N5—C16	1.381 (4)	C17—H17	0.9300
N5—C12	1.386 (4)	C18—C19	1.371 (6)
N5—H5B	0.8600	C18—H18	0.9300
N6—C16	1.332 (4)	C19—C20	1.350 (6)
N6—C20	1.342 (4)	C19—H19	0.9300
C1—C2	1.392 (4)	C20—H20	0.9300
C1—H1	0.9300	Cl1—O4	1.377 (4)
C2—C3	1.388 (4)	Cl1—O1	1.397 (3)
C3—C4	1.362 (5)	Cl1—O3	1.404 (3)
C3—H3	0.9300	Cl1—O2	1.415 (3)
C4—C5	1.381 (4)	O5—C22	1.368 (6)
C4—H4	0.9300	O5—H5A	0.8200
C5—H5	0.9300	C21—C22	1.397 (7)
C6—C7	1.394 (5)	C21—H21A	0.9600
C7—C8	1.376 (5)	C21—H21B	0.9600
C7—H7	0.9300	C21—H21C	0.9600
C8—C9	1.369 (5)	C22—H22A	0.9700
C8—H8	0.9300	C22—H22B	0.9700
			
N1^i^—Ni1—N1	89.08 (14)	N4—C11—C12	123.5 (3)
N1^i^—Ni1—N4	179.09 (11)	N4—C11—H11	118.2
N1—Ni1—N4	90.24 (10)	C12—C11—H11	118.2
N1^i^—Ni1—N4^i^	90.24 (10)	C13—C12—C11	117.5 (3)
N1—Ni1—N4^i^	179.09 (11)	C13—C12—N5	124.7 (3)
N4—Ni1—N4^i^	90.44 (13)	C11—C12—N5	117.7 (3)
C1—N1—C5	119.3 (3)	C12—C13—C14	119.3 (3)
C1—N1—Ni1	120.49 (19)	C12—C13—H13	120.4
C5—N1—Ni1	120.0 (2)	C14—C13—H13	120.4
C6—N2—C2	128.5 (3)	C15—C14—C13	120.0 (3)
C6—N2—H2	115.7	C15—C14—H14	120.0
C2—N2—H2	115.7	C13—C14—H14	120.0
C6—N3—C10	117.1 (3)	N4—C15—C14	121.4 (3)
C11—N4—C15	118.2 (3)	N4—C15—H15	119.3
C11—N4—Ni1	121.5 (2)	C14—C15—H15	119.3
C15—N4—Ni1	120.2 (2)	N6—C16—N5	118.6 (3)
C16—N5—C12	127.8 (3)	N6—C16—C17	122.7 (3)
C16—N5—H5B	116.1	N5—C16—C17	118.7 (3)
C12—N5—H5B	116.1	C18—C17—C16	118.4 (4)
C16—N6—C20	116.6 (3)	C18—C17—H17	120.8
N1—C1—C2	122.8 (3)	C16—C17—H17	120.8
N1—C1—H1	118.6	C17—C18—C19	119.6 (4)
C2—C1—H1	118.6	C17—C18—H18	120.2
C3—C2—C1	117.0 (3)	C19—C18—H18	120.2
C3—C2—N2	118.7 (3)	C20—C19—C18	118.0 (4)
C1—C2—N2	124.3 (3)	C20—C19—H19	121.0
C4—C3—C2	120.2 (3)	C18—C19—H19	121.0
C4—C3—H3	119.9	N6—C20—C19	124.7 (4)
C2—C3—H3	119.9	N6—C20—H20	117.7
C3—C4—C5	119.8 (3)	C19—C20—H20	117.7
C3—C4—H4	120.1	O4—Cl1—O1	109.0 (3)
C5—C4—H4	120.1	O4—Cl1—O3	109.4 (3)
N1—C5—C4	120.9 (3)	O1—Cl1—O3	108.6 (2)
N1—C5—H5	119.6	O4—Cl1—O2	111.8 (3)
C4—C5—H5	119.6	O1—Cl1—O2	107.7 (2)
N3—C6—N2	118.7 (3)	O3—Cl1—O2	110.3 (2)
N3—C6—C7	122.8 (3)	C22—O5—H5A	109.5
N2—C6—C7	118.5 (3)	C22—C21—H21A	109.5
C8—C7—C6	118.0 (4)	C22—C21—H21B	109.5
C8—C7—H7	121.0	H21A—C21—H21B	109.5
C6—C7—H7	121.0	C22—C21—H21C	109.5
C9—C8—C7	119.5 (4)	H21A—C21—H21C	109.5
C9—C8—H8	120.3	H21B—C21—H21C	109.5
C7—C8—H8	120.3	O5—C22—C21	118.1 (6)
C10—C9—C8	118.4 (4)	O5—C22—H22A	107.8
C10—C9—H9	120.8	C21—C22—H22A	107.8
C8—C9—H9	120.8	O5—C22—H22B	107.8
N3—C10—C9	124.2 (4)	C21—C22—H22B	107.8
N3—C10—H10	117.9	H22A—C22—H22B	107.1
C9—C10—H10	117.9		
			
N1^i^—Ni1—N1—C1	−48.8 (2)	C6—C7—C8—C9	−1.2 (6)
N4—Ni1—N1—C1	130.6 (2)	C7—C8—C9—C10	1.0 (6)
N1^i^—Ni1—N1—C5	125.6 (3)	C6—N3—C10—C9	−1.8 (6)
N4—Ni1—N1—C5	−55.0 (3)	C8—C9—C10—N3	0.5 (6)
N1—Ni1—N4—C11	−59.8 (2)	C15—N4—C11—C12	−0.2 (4)
N4^i^—Ni1—N4—C11	120.8 (3)	Ni1—N4—C11—C12	175.7 (2)
N1—Ni1—N4—C15	116.0 (2)	N4—C11—C12—C13	−3.4 (4)
N4^i^—Ni1—N4—C15	−63.4 (2)	N4—C11—C12—N5	179.0 (3)
C5—N1—C1—C2	−1.2 (5)	C16—N5—C12—C13	25.5 (5)
Ni1—N1—C1—C2	173.2 (2)	C16—N5—C12—C11	−157.0 (3)
N1—C1—C2—C3	1.7 (5)	C11—C12—C13—C14	4.5 (4)
N1—C1—C2—N2	−179.5 (3)	N5—C12—C13—C14	−178.0 (3)
C6—N2—C2—C3	−157.8 (3)	C12—C13—C14—C15	−2.2 (5)
C6—N2—C2—C1	23.3 (6)	C11—N4—C15—C14	2.7 (4)
C1—C2—C3—C4	−0.9 (5)	Ni1—N4—C15—C14	−173.3 (2)
N2—C2—C3—C4	−179.9 (3)	C13—C14—C15—N4	−1.5 (5)
C2—C3—C4—C5	−0.2 (5)	C20—N6—C16—N5	−179.7 (3)
C1—N1—C5—C4	0.0 (5)	C20—N6—C16—C17	2.8 (5)
Ni1—N1—C5—C4	−174.4 (2)	C12—N5—C16—N6	7.6 (5)
C3—C4—C5—N1	0.6 (5)	C12—N5—C16—C17	−174.8 (3)
C10—N3—C6—N2	179.8 (3)	N6—C16—C17—C18	−1.5 (5)
C10—N3—C6—C7	1.5 (5)	N5—C16—C17—C18	−179.0 (3)
C2—N2—C6—N3	1.4 (6)	C16—C17—C18—C19	−0.9 (6)
C2—N2—C6—C7	179.8 (3)	C17—C18—C19—C20	1.8 (7)
N3—C6—C7—C8	−0.1 (6)	C16—N6—C20—C19	−1.9 (6)
N2—C6—C7—C8	−178.4 (3)	C18—C19—C20—N6	−0.4 (7)

Symmetry codes: (i) −*x*+1, *y*, −*z*+3/2.

### Hydrogen-bond geometry (Å, °)

**Table:**

*D*—H···*A*	*D*—H	H···*A*	*D*···*A*	*D*—H···*A*
N5—H5B···O5	0.86	2.07	2.928 (5)	174
N2—H2···N6^ii^	0.86	2.27	3.129 (4)	176

Symmetry codes: (ii) *x*, *y*−1, *z*.
